# The evolution of alternative splicing in glioblastoma under therapy

**DOI:** 10.1186/s13059-021-02259-5

**Published:** 2021-01-26

**Authors:** Lin Wang, Karin Shamardani, Husam Babikir, Francisca Catalan, Takahide Nejo, Susan Chang, Joanna J. Phillips, Hideho Okada, Aaron A. Diaz

**Affiliations:** 1 Department of Neurological Surgery, University of California, San Francisco, 1450 3rd Street, San Francisco, CA 94158 USA; 2 Helen Diller Family Comprehensive Cancer Center, 1450, 3rd Street, San Francisco, CA 94158 USA; 3 Parker Institute for Cancer Immunotherapy , 1 Letterman Dr Suite D3500, Building D, San Francisco, CA 94129 USA

## Abstract

**Background:**

Alternative splicing is a rich source of tumor-specific neoantigen targets for immunotherapy. This holds promise for glioblastomas (GBMs), the most common primary tumors of the adult brain, which are resistant to standard-of-care therapy. Although most clinical trials enroll patients at recurrence, most preclinical studies have been done with specimens from primary disease. There are limited expression data from GBMs at recurrence and surprisingly little is known about the evolution of splicing patterns under therapy.

**Result:**

We profile 37 primary-recurrent paired human GBM specimens via RNA sequencing. We describe the landscape of alternative splicing in GBM at recurrence and contrast that to primary and non-malignant brain-tissue specimens. By screening single-cell atlases, we identify cell-type-specific splicing patterns and novel splicing events in cell-surface proteins that are suitable targets for engineered T cell therapies. We identify recurrent-specific isoforms of mitogen-activated kinase pathway genes that enhance invasiveness and are preferentially expressed by stem-like cells.

**Conclusion:**

These studies shed light on gene expression in recurrent GBM and identify novel targets for therapeutic development.

**Supplementary Information:**

The online version contains supplementary material available at 10.1186/s13059-021-02259-5.

## Introduction

Alternative-splicing (AS) events have recently been identified as a source of neoantigens that are suitable for immunotherapy (e.g., [1]). This observation has greatly increased the scope of neoantigen targets. For example, over 68% of breast and ovarian cancers express an AS-derived neoepitope while only 30% of cases express a neoepitope derived from a single-nucleotide variant (SNV) [2]. Moreover, AS drives a variety of malignant phenotypes including invasiveness, angiogenesis, and aberrant metabolism [3]. Thus, mapping AS is of high clinical importance, to identify novel therapeutic and predictive biomarkers of malignant progression.

Surprisingly, little is known about gene expression in recurrences from primary glioblastomas (GBMs), despite GBM being the most common and most deadly primary adult-brain tumors. The majority of GBM preclinical studies have relied on models of primary disease and/or tissues from primary GBMs. This is a significant caveat that we address by profiling paired primary and recurrent human GBM specimens.

We combined de novo RNA sequencing (RNA-seq) of paired GBM clinical specimens with public RNA-seq of malignant and non-malignant, adult and fetal brain tissues to construct an integrated model of AS during GBM malignant progression. We screened the AS events we detected in single-cell RNA-seq (scRNA-seq) data from human GBM specimens to determine their cell-type specificity. We identified an exon-retention event upregulated in recurrent GBM in mitogen-activated protein 4 kinase 4 (MAP4K4), and we inferred serine- and arginine-rich splicing factor 5 (SRSF5) as an upstream regulator. This alteration is specific to stem-like cells of the Verhaak mesenchymal phenotype and has been previously implicated in c-Jun N-terminal kinase (MAPK8)-dependent invasion. We overexpressed SRSF5 in a GBM patient-derived cell line and found it enhanced invasion of extracellular matrix (ECM).

Chimeric-antigen-receptor (CAR) T cells are a novel engineered T cell approach, where donor T cells can be programmed to engage cytotoxic function when triggered by an antigen target. Ideal targets are cell-surface proteins specifically altered in tumor cells compared to non-malignant glia, leukocytes, or endothelial cells, to minimize off-target effects. We leveraged a combination of bulk RNA-seq and scRNA-seq to screen for AS events that alter extracellular domains specifically in GBM neoplastic cells. Additionally, we screened tumor-specific AS events for their potential to be processed and presented by class-I human leukocyte antigen, thus making them available as targets for T cell receptor-transduced therapy or cancer vaccine development.

We conclude that (1) specific AS events and splicing factors are enriched in GBM at recurrence, (2) *SRSF5* is upregulated in recurrent GBM and promotes glioma invasion, and (3) many highly tumor-specific AS events are prevalent in the GBM population and are promising candidates for autologous T cell approaches. These studies enhance our understanding of the progression of AS in GBM at recurrence and elucidate novel potential targets for immunotherapy.

## Results

### Profiling AS in GBM through recurrence

**Fig. 1:**
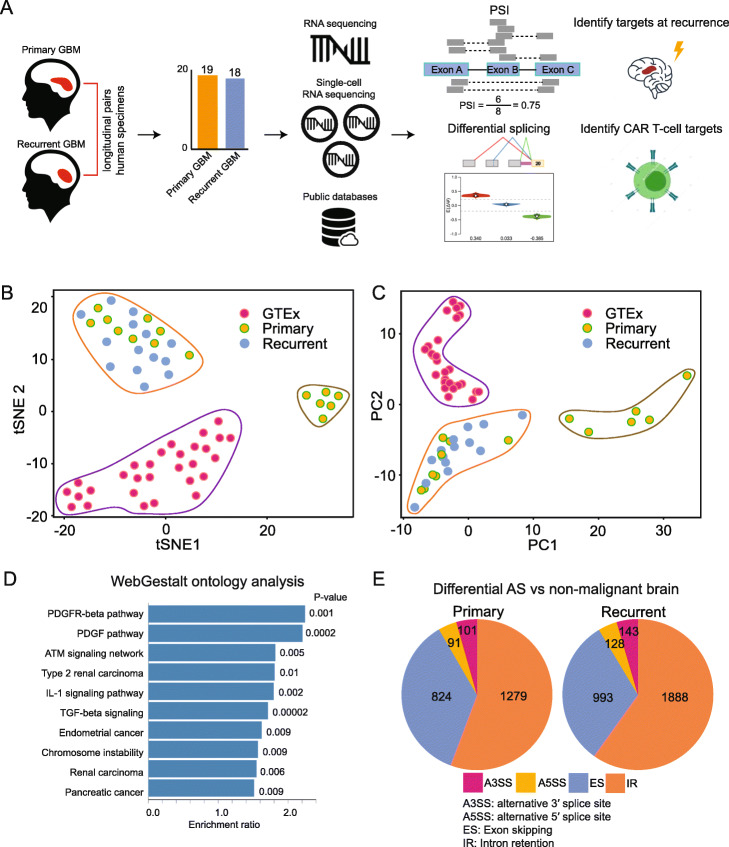
**a** Conceptual overview of study design and samples used. **b** T-distributed stochastic neighbor (tSNE) embedding of AS PSI across primary GBM, recurrent GBM, and GTEx non-malignant brain samples. **c** As in **b**, but PCA. **d** Wikipathway Cancer gene-ontology analysis of genes within the top 20% loadings of positive and negative principal component one from **c**. **e** The distributions of AS types in tumor-specific AS events, comparing primary and recurrent GBMs

We profiled 37 human GBM specimens from 23 patients, 19 primary untreated cases, and 18 recurrent cases treated with standard-of-care therapy (radiation, temozolomide, and surgical resection); 34 specimens were patient-matched longitudinal samples (Fig. 1a; Additional file 1: Table S1). We performed RNA-seq on each of these specimens, generating over 277 million reads per sample. Additionally, we obtained 15 public RNA-seq datasets from longitudinal GBM specimens and 29 public RNA-seq datasets from non-malignant adult and fetal brain tissues (“Materials and methods” section).

We constructed an integrated model of AS between non-malignant brain, primary and recurrent GBM conditions via MAJIQ [4] (Fig. 1b; “Materials and methods” section). We found that non-malignant brain samples formed a distinct cluster, separating from primary and recurrent GBM specimens, when viewed in a principal-components analysis (PCA) of marginal percent-selected indices (PSIs) (Fig. 1c; Additional file 2: Table S2). The PSI values were computed via MAJIQ’s Bayesian model and estimate the frequencies with which splice junctions are selected in AS events. A gene-ontology analysis of AS events whose PSIs variably loaded principal component one revealed growth-factor signaling (e.g., *PDGFRB*, *PIK3R1*, *FOS*, *MAPK9*), tumor-growth factor (e.g., *TGFB1*, *SMAD2*, *SMAD4*), and pro-inflammatory (e.g., *NFKB1*, *RELA*, *STAT1*, *STAT3*) signaling (Fig. 1d, Additional file 3: Fig. S1). The relative proportions of AS event types were stable when comparing primary and recurrent disease (Fig. 1e).

### Identification of targets for autologous T cell therapy

**Fig. 2:**
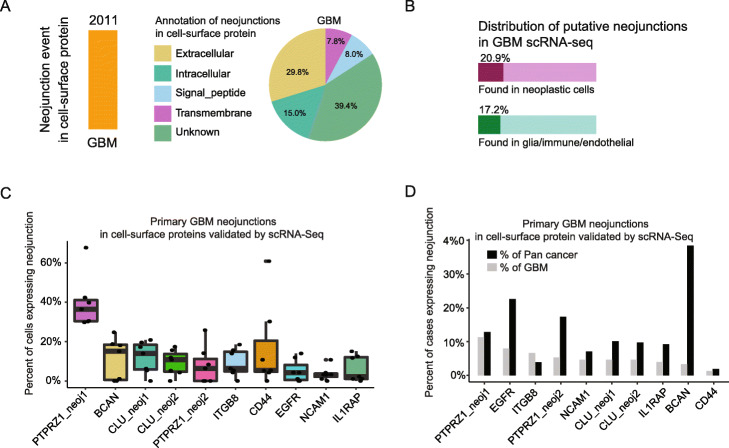
**a** The frequencies of neojunctions identified by Kahles et al. [2] across the domains of cell-surface proteins. **b** The frequencies of neojunctions in the extracellular domains of cell-surface proteins and in neoplastic cells vs. non-malignant glia, immune, and endothelial cells. **c** The frequencies of expression of neojunction sequences in human GBM neoplastic cells in Smart-seq2 scRNA-seq data. **d** The frequencies of neojunctions across the GBM and pan-cancer populations, as assessed from The Cancer Genome Atlas (TCGA) RNA-seq data

We began by screening for GBM-specific targets in cancer-specific neojunctions that had been previously identified in a pan-cancer analysis [2]. We identified 2011 putative neojunction events in cell-surface proteins expressed in GBMs. Of these, 37.8% fell in extracellular domains and would therefore be suitable as CAR T cell targets (Fig. 2a). We then compared human GBM scRNA-seq data (“Materials and methods” section), to validate neojunction sequences as being expressed in neoplastic cells, but not expressed in non-malignant glia or immune cells (Fig. 2b). We found a variety of neojunctions that are specifically expressed by GBM neoplastic cells (Additional file 4: Table S3). These included extracellular matrix receptors long-studied as mediators of GBM invasion (e.g., *PTPRZ1*; Fig. 2c) [5], as well as the marker of glioma stem cells of the Verhaak mesenchymal subtype, *CD44*. We found several target sequences expressed in 10–35% of neoplastic cells within individual tumors and across 5–10% of GBM cases (Fig. 2d, Additional file 3: Fig. S2A).

**Fig. 3:**
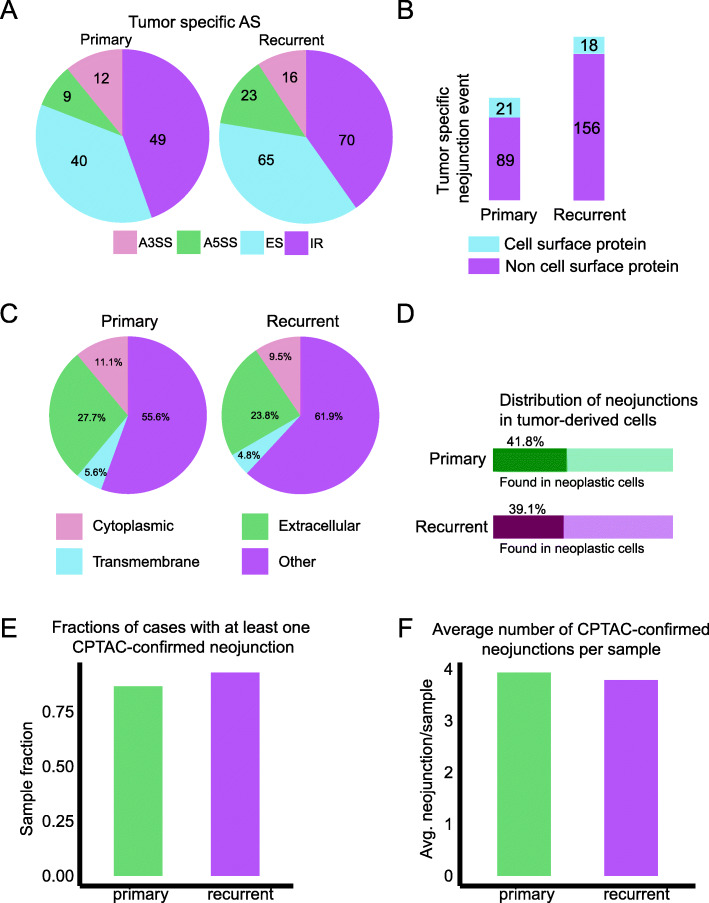
**a** The distribution of AS event type in tumor-specific AS events, comparing primary and recurrent GBMs. **b** The fractions of tumor-specific AS events in cell-surface proteins, compared between primary and recurrent GBM. **c** The percentages of tumor-specific neojunctions found in different protein domains. **d** The frequencies of tumor-specific neojunctions in GBM neoplastic cells from Smart-seq2 scRNA-seq. **e** The fractions of cases with at least one CPTAC-confirmed neojunction. **f** The average number of CPTAC-confirmed neojunctions per case

We then interrogated our novel RNA-seq data for tumor-specific AS events. We identified differentially spliced genes between all GBM samples vs. non-malignant brain samples (“Materials and methods” section; Additional file 5: Table S4). These events were then filtered to retain only those that were completely absent in the non-malignant brain. To that end, we only further considered AS events with PSI = 0 in all non-malignant brain samples and expected absolute PSI > 10% at a 95% confidence level in tumor specimens. We found far fewer tumor-specific events than in our previous differential splicing analysis (Fig. 3a). Nonetheless, we identified 221 tumor-specific AS events (Fig. 3b), with the majority occurring only in recurrent GBM (Additional file 3: Fig. S2B). When we compared our GBM scRNA-seq data, we found there were 21 and 18 putative neojunction events in cell-surface proteins that were specifically expressed in neoplastic cells and were present in extracellular domains (Fig. 3c, d; Additional file 6: Table S5). Following the approach of Kahles et al. [2], we derived neojunction-spanning polypeptide sequences and compared them to the Clinical Proteomic Tumor Analysis Consortium (CPTAC) database [6]. We found that over 75% of our samples expressed at least one CPTAC-confirmed neojunction (Fig. 3e), with three-to-four neojunctions confirmed per sample on average (Fig. 3f). We consider this to be a conservative underestimate since alternative splicing-derived junction-spanning polypeptides are poorly represented in mass-spectrometry data such as CPTAC due to the cleavage properties of trypsin [7]. Thus, all of our RNA-level candidates would be suitable for further validation and development as CAR T cell targets.

**Fig. 4:**
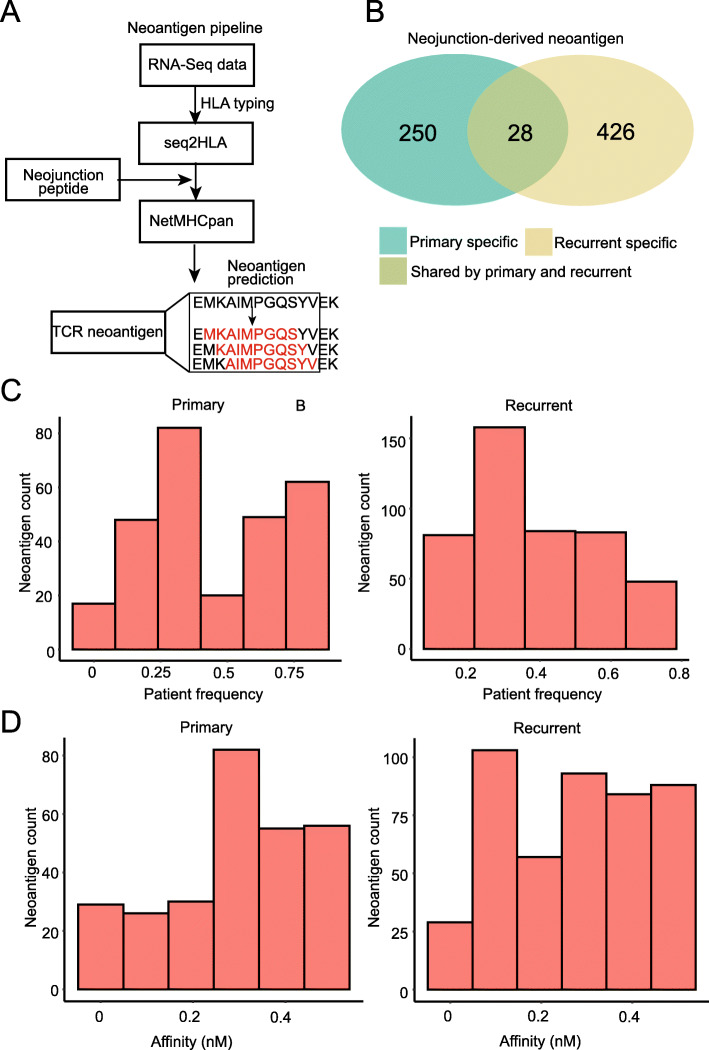
**a** An overview of the neoantigen discovery pipeline. **b** The numbers of primary-tumor enriched, recurrent-tumor-enriched, and shared putative neojunction-derived neoantigens. **c** The frequencies of neojunction-derived neoantigens in primary vs. recurrent GBM. **d** The HLA-I predicted binding affinities of neojunction-derived neoantigens in primary vs. recurrent GBM

Although our primary focus was the identification of targets for CAR T cells, we also screened for putative targets for T cell receptor (TCR)-transduced T cell therapies. TCR T cells are less flexible than CAR T cells, in that they require target processing and presentation on class-I human leukocyte antigen (HLA). On the other hand, CAR T cells can target any cell-surface protein regardless of HLA presentation and peptide processing is not a prerequisite. Thus, to identify putative targets for TCR T cell therapy, we first needed to determine the class-I HLA serotype for each patient from the associated RNA-seq data (Fig. 4a; “Materials and methods” section; Additional file 7: Table S6). We then extracted sequences from the reference in a 50 base-pair window around each of our putative neojunctions and used NetMHCpan to predict cleaved peptides from the associated protein product. NetMHCpan was further used to predict HLA binding of generated peptides, given the patient’s serotype. We identified 704 neojunction-derived putative neoantigens (Fig. 4b). Note that a single neojunction may lead to multiple putative neoantigens. We observed an increase both in the number of neoantigens inferred and in the predicted binding affinity of those antigens in recurrent GBMs (Fig. 4c, d).

### AS events enriched in recurrent GBM

**Fig. 5:**
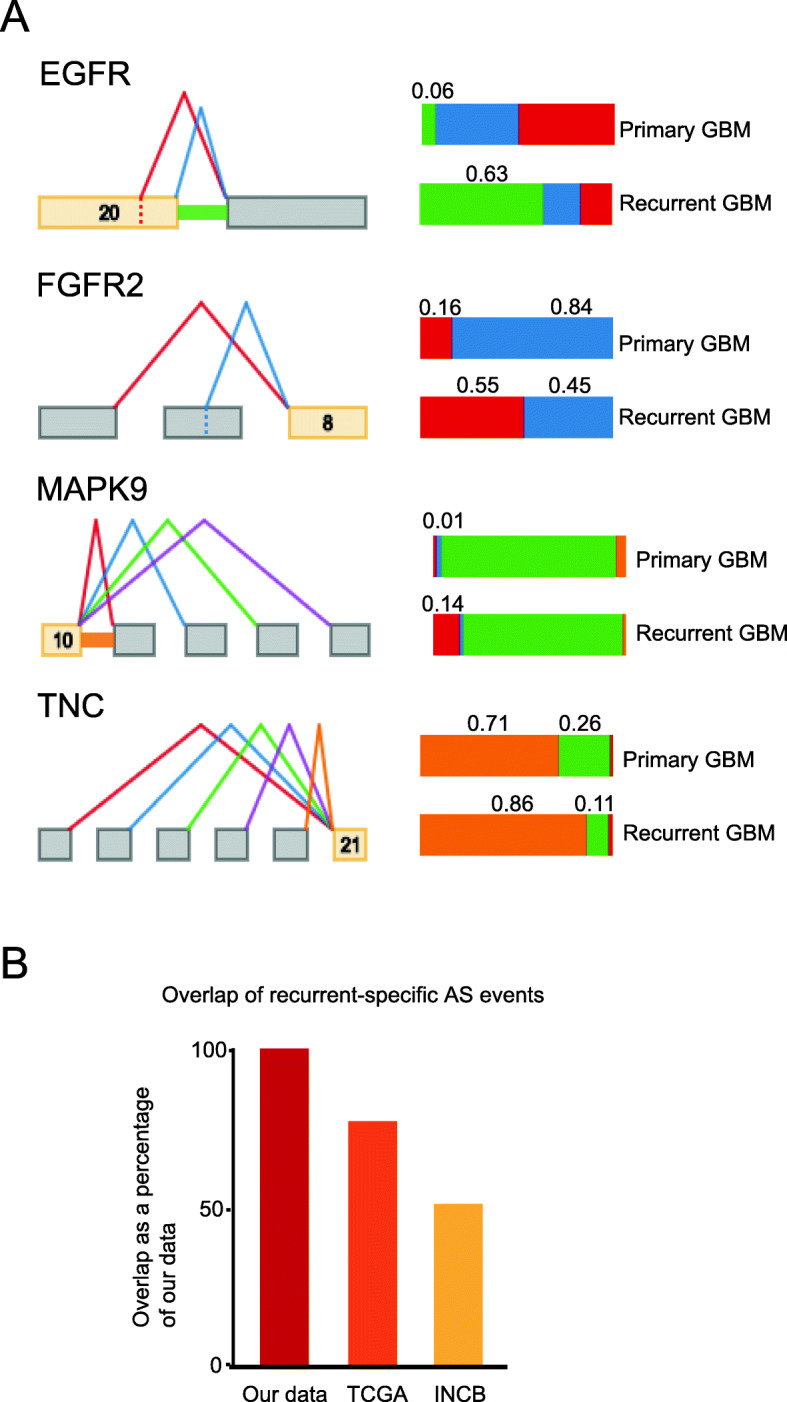
**a** Examples of AS events with significant differential splicing between primary and recurrent GBMs. **b** The percentages of overlap between recurrent-specific AS events in our data, compared to recurrent-specific expression in TCGA and INCB data

Next, we performed a differential PSI test via MAJIQ, comparing primary and recurrent specimens. We identified 172 AS events in 107 genes (100 coding and 7 long non-coding RNA) with an expected differential PSI greater than 10%, at the 95% confidence level (“Materials and methods” section; Additional file 8: Table S7). Many of these events occurred in genes that are critical for malignant progression. For example, several mitogen-activated protein kinases (*MAP4K4*, *MAPK9*, *MAPK10*), growth-factor receptors (*FGFR1*, *FGFR2*, *EGFR*), and matricellular proteins (*TNC*, *FN1*) showed significant differences in PSI between primary and recurrent GBM (Fig. 5a, Additional file 3: Fig. S3A). We found that these and other AS which were enriched in recurrent GBMs in our data were also enriched in recurrent cases in publicly available GBM RNA-seq [8] (Fig. 5b).

### Recurrent GBMs preferentially express isoforms that enhance invasion

**Fig. 6:**
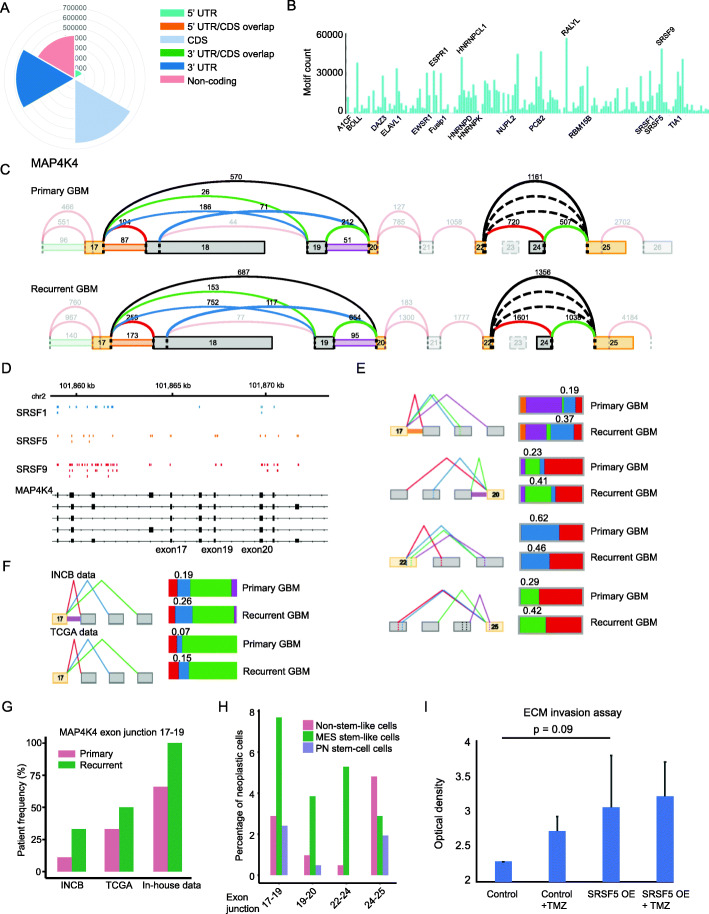
**a** The distribution of RNA-binding-protein recognition sites across differentially spliced genes between primary and recurrent GBM. **b** The frequencies of occurrences of RNA-binding protein motifs in differentially spliced genes between primary and recurrent GBM. **c** Alternative exon inclusion in *MAP4K4*. **d** Inferred binding sites for SRSF proteins in *MAP4K4.* **e** PSI values for junctions supporting exon 19 inclusion and others, compared between primary and recurrent GBM RNA-seq, using the in-house data. **f** As in **e**, but using public data from TCGA and INCB. **g** The frequencies of expression of exon-19 supporting neojunction sequences in primary vs. recurrent GBM, compared between our in-house data, TCGA and INCB data. **h** The frequencies of occurrence of *MAP4K4* exon junctions supporting exon-19 inclusion in stem-like and non-stem-like neoplastic GBM cells. **i** The results of an extracellular matrix invasion assay comparing SRSF5 OE in U87 cells to controls, with and without TMZ treatment at the IC50 for 48 h

The sequences of differentially spliced genes were scanned for RNA-binding protein motifs and putative binding sites. The majority of binding sites were found in coding regions, fewer in untranslated and intronic regions (Fig. 6a). Among the most frequently observed motifs were those recognized by previously described trans-mediators of AS. In particular, several serine- and arginine-rich splicing factor (SRSF) RNA-binding proteins were identified (Fig. 6b). SRSF splicing factors have been previously implicated in cancer progression for their ability to bind variable exons and inhibit or promote exon skipping (e.g., [9]).

Some predicted SRSF binding sites occurred in exons that were specifically retained in recurrent GBM. For example, MAP4K4 selectively interacts with MAPK8 to promote migration and invasion in cancer, dependent on the inclusion of exon 19 [10, 11]. A recent loss-of-function screen identified *MAP4K4* as essential for GBM invasion and epithelial-to-mesenchymal transition [12]. We found exon 19 of *MAP4K4* to be preferentially retained in recurrent GBM in our data, but preferentially spliced out in primary GBM (Fig. 6c). Moreover, SRSF5 and SRSF9 motifs (two of the most overrepresented in our analysis) were enriched in exon 19 (Fig. 6d). This enrichment for exon 19 inclusion at recurrence was pronounced in our data (Fig. 6e) and validated in public longitudinal GBM RNA-seq data [8], both via MAJIQ modeling (Fig. 6f) and in terms of the number of primary vs. recurrent cases expressing the associated neojunction (Fig. 6g).

To determine the cell-type specificity of *MAP4K4* isoform expression, we screened published scRNA-seq data that were obtained via the Smart-seq2 and 10X Genomics platforms [13, 14]. We found an over 2-fold increase in exon-19-supporting junction sequences in stem-like cells compared to cells with a more differentiated phenotype (Fig. 6h, Additional file 3: Fig. S3B; “Materials and methods” section). Moreover, this isoform was found predominantly in stem-like cells of the Verhaak mesenchymal subtype. Consistent with this isoform’s role in stimulating MAPK8, we found significantly increased *MAPK8* expression in recurrent GBM (adj. *p* = 0.045; Additional file 9: Table S8).

To determine the effect of *SRSF5* overexpression (OE), we transfected the patient-derived glioma cell line U87 with plasmids expressing *SRSF5* or empty-vector controls (“Materials and methods” section). Transfected cells were selected via flow cytometry using a fluorescent marker expressed by the vector; 500,000 cells per condition (in duplicate) were aliquoted for a Boyden-chamber-based extracellular matrix invasion assay. We considered two arms, with and without 48-h treatment with GBM standard-care therapy temozolomide (TMZ) at half the maximal inhibitory concentration (IC50), which we had determined from previous studies for this cell line [14]. We found that *SRSF5* OE increased invasiveness and that this effect was exacerbated by TMZ treatment (Fig. 6i).

## Discussion

Dysregulation of the splicing machinery is the hallmark of several malignancies, including glioma [2]. In particular, aberrant SRSF expression is associated with malignant progression in a variety of cancers and some SRSFs are proto-oncogenes (e.g., [15–17]). SRSF3 was recently shown to positively regulate the tumorigenicity of GBM cells [18]. We found that the retention of exon 19 of *MAP4K4* correlated with SRSF5 and SRSF9 binding sites in recurrent GBM. This was concomitant with the upregulation of MAPK8 at recurrence, and specific to GBM stem-like cells of the Verhaak mesenchymal phenotype (Fig. 6h). Retention of exon 19 in MAP4K4 is associated with invasiveness in colorectal cancer [11], and MAP4K4 is essential for GBM invasion [12]. Intriguingly, we find both SRSF5 and SRSF9 increased in GBM at recurrence, along with MAPK8 (Additional file 9: Table S8). We found that *SRSF5* OE enhanced GBM invasion into extracellular matrix in vitro and that this effect was exacerbated by TMZ treatment.

CAR T cell therapy is an emerging platform with promise for solid tumors that have a high degree of local immunosuppression, such as gliomas of the brain [19]. CAR T cells overcome several limitations of TCR T cell approaches, such as the need for HLA expression, HLA identity, and co-stimulation. Moreover, loss of HLA expression and HLA-associated neoantigen expression are common mechanisms of immune evasion by cancer cells. Thus, CAR T cell approaches have a significant advantage over TCR T cell strategies since they do not depend on HLA expression and neoantigen display. Rather CAR T cells can target any cell-surface protein for which there is an antibody. However, a limiting factor in the development of engineered T cells is a lack of suitable targets. We identified several novel targets for CAR T cell development in GBM. We found that there are novel putative CAR targets in recurrent GBM that are not present in primary GBM. Moreover, we identified multiple neojunction-derived neoantigens that are putative targets for TCR T cell approaches.

Caveats of this study include its focus on expression at the mRNA level. Further work will be needed to characterize these targets at the protein level. Additional studies will be required to identify antibodies that recognize these targets specifically, and to develop and test CAR T cell reagents. Sample size has also been a limitation for longitudinal studies of RNA expression in human GBMs, as recurrent GBMs are not always biopsied and where archival tissue is available for research purposes RNA is often degraded, especially in formalin-treated specimens. The novel cohort presented here is the result of decades of biobanking at our institution. These studies show that there are multiple AS-derived targets for autologous T cell therapy which are expressed broadly in GBM and other cancers (Fig. 2d). Thus, this study has produced a resource for the development of immunotherapies with broad application. These studies elucidate AS and gene expression in the understudied context of recurrent GBM.

## Materials and methods

### Tumor tissue acquisition

We acquired formalin-fixed paraffin-embedded (FFPE) tissue from patients undergoing surgical resection for glioma at UCSF. De-identified samples were provided by the UCSF Neurosurgery Tissue Bank. Sample use was approved by the Institutional Review Board at UCSF. The experiments performed here conform to the principles set out in the WMA Declaration of Helsinki and the Department of Health and Human Services Belmont Report. All patients provided informed written consent.

### Tissue processing and de novo RNA-seq

FFPE blocks were reviewed by a pathologist to identify regions of high tumor purity. Total RNA was purified from freshly punched cores via a Qiagen RNeasy FFPE purification column. RNA and DNA were quantified via both Bioanalyzer and Qubit to ensure sample quality. All samples yielded over 100 ng RNA with DV200 > 30% and negligible DNA contamination. For library prep, we used the Illumina TruSeq RNA Exome kit according to the manufacturer’s instructions. This approach enriches for coding sequences with a capture array and is suitable for fragmented RNA extracted from FFPE tissue.

Paired-end sequencing was done on the Illumina NovaSeq platform yielding over 277 million read-pairs per sample. Sequenced reads were trimmed by using Trim Galore to trim sequences with base-call Phred score < 30 and to remove Illumina adapter sequences (cutadapt version 1.2.1 parameters: -f fastq -e 0.1 -q 30 -O 1 --illumina). Over 99% reads did not require trimming. Trimmed reads were aligned with HISAT2 [20] to grCh38. The parameter “--no-unal” of HISAT2 was applied, and other parameters were set as default when we did the alignment. Only correctly paired, uniquely mapped reads were retained for further analysis. More than 96.3% of the reads per sample satisfied this criterion. Gene expression was quantified using the ENSEMBL reference (release 25) with featureCounts [21]. Only correctly paired, uniquely mapped reads were retained.

DESeq2 (likelihood ratio test) was applied to perform a differential expression test between primary and recurrent cases, using the read counts generated by featureCount. The Fdrtool package (version 1.2.1) [22] was used to adjust *p* values for multiple hypothesis testing. Genes with adjusted *p* values less than 0.05 were considered significantly differentially expressed.

### Alternate-splicing analyses

Alternative-splicing analysis was performed using MAJIQ (version 2.1) and VOILA (version 2.0) [4]. MAJIQ-build was used to define and quantify a splice graph of known and novel local splice variations (LSV). An ENSEMBL (release 25) GFF3 reference file was used as input to define known LSVs. The parameters for MAJIQ-build were set to be --min-experiments > 0.5, --min-intronic-cov (minimum number of reads on average in intronic sites) > 0.01, --min-denovo (minimum number of reads threshold combining all positions in a LSV to consider that denovo junction is real) > 5 on the default minimum number of positions = 3.

MAJIQ-Deltapsi was used to identify differential alternative splicing events. In particular, the following parameters were applied: --min-experiments > 0.5, --prior-minreads (minimum number of reads combining all positions in a junction to be considered) > 20, and --minreads (minimum number of reads combining all positions in an event to be considered) > 10 on the default minimum number of positions = 3. VOILA was used to summarize and visualize MAJIQ output with –threshold value > 0.1. Differential splicing events were identified at a threshold of abs(E (dPSI)) > 0.1 at the 95% confidence level.

### Other bioinformatics analyses

Exon-skipping events were used for PCA analysis and included at a threshold of *E* (PSI) > 0.1. Tumor-specific splicing events were obtained by thresholding PSI = 0 in the GTEx non-malignant brain data. RNA-binding protein motif enrichment was done via oRNAment [23].

WebGestalt [24] was used to perform gene-ontology term overrepresentation analysis via the “Wikipathway cancer” database. A genome-wide background was used and the “minimum number of IDs in the category” was set to 5. Genes with AS events within the top 20% largest loadings of positive and negative principle components 1 were combined and used as an input gene list for WebGestalt.

ScRNA-seq data from 9 human GBMs (4 Smart-seq2 and 5 10X Genomics datasets, 7859 cells in total) were obtained from [13, 14]. Stemness scores were calculated via the AddModuleScore function from the Seurat package (version 3) [25], using gene signatures previously described [14]. Cell-type classification of scRNA-seq was done via ELSA [26], using gene signatures previously identified [14, 27].

BLASTn was used to map sequences from scRNA-seq, TCGA, and INCB datasets to AS-junction-derived references in order to validate neojunctions and assess their cell-type specificity. For this purpose, references were constructed to include 600 bp of sequence from the grCh38 reference in a symmetric 300-bp interval around AS exon-exon junctions.

Mass-spectrometry data were obtained from CPTAC Data Portal (https://cptac-data-portal.georgetown.edu/). Neojunction-derived polypeptides were derived by translating nucleotide sequences from the grCh38 reference genome, considering three reading frames. Nucleotide sequences of 48 base pairs, symmetrically centered at each of the splice junctions, were used as input. These neojunction-derived neopeptides were then compared to the GBM mass-spectrometry data from CPTAC. This dataset was generated via tandem mass-spectrometry applied to 11 analytical samples, comprised of protein extractions pooled from 10 patient samples each. The CPTAC-preferred software suite, OpenMS, was used to perform polypeptide screening. Briefly, decoy sequences were generated and added to the query database via the DecoyDatabase tool. MSGFPlusAdapter was then used to search the CPTAC data for query sequences. Hits were then filtered via IDFilter based on a false-discovery rate of 0.05, as estimated from decoy-sequence hits.

RNA-seq of non-malignant human brain tissues were obtained from the GTEx portal (https://www.gtexportal.org/home/datasets). Cell-surface protein and protein-domain annotations were obtained from the Cell-Surface Protein Atlas (http://wlab.ethz.ch/cspa) and Uniprot.org (via the UCSC Genome browser https://genome.ucsc.edu) respectively. The paired longitudinal GBM data from TCGA were obtained from Genomic Data Commons Portal (https://portal.gdc.cancer.gov/), and those from INCB were downloaded from the Sequence Read Archive (SRP074425). The alignment and alternative splicing analysis of TCGA data and INCB data were processed as above.

### Neoantigen prediction

We used seq2HLA (version 2.2) to infer patients’ HLA class I serotype from the RNA-seq data. This approach aligns the RNA-Seq reads against a reference database of HLA alleles and determines the HLA type, confidence score, and locus-specific expression level for each class. To obtain neojunction-derived sequences, 50 base pairs of sequence was extracted from the reference around the neojunction coordinate. NetMHCpan (version 3.0) was then run with default parameters to predict cleaved peptides and HLA binding affinity, using neojunction-derived sequences and patient-specific HLA serotypes as input. We only kept strong binding neoantigens from the output of NetMHCpan, defined as having a percent rank < 0.5 of the predicted affinity compared to a set of 400,000 random natural peptides.

### Overexpression of SRSF5 in the U87 glioma cell line

The patient-derived GBM cell line U87-MG was authenticated via RNA and exome sequencing prior to use. U87-MG cells were transfected either with SRSF5-GFP plasmid (Origene CAT#: RC218652L2) or with control-GFP plasmid (Origene CAT#: PS100010) using FuGENE transfection reagent (Promega, Catalog number E2311). Flow cytometry was employed to sort populations of cells according to equivalent levels of GFP marker expression. Cells were maintained in DMEM media supplemented with 10% FBS and 100 U/mL penicillin and 0.1% streptomycin.

### Cell invasion assay

GFP-labeled cells cultured in DMEM media were treated with either 125 μM of temozolomide or DMSO control for 48 h. Cells were trypsinized and 500,000 of each condition were plated in serum-free medium in the upper chambers of ECMatrix invasion chambers with coated polycarbonate membranes (24-well insert, 8 μm pore size—ECM550) (Millipore, Billerica, MA); medium supplemented with 10% FBS was added to the lower chambers. Cells were then incubated for an additional 24 h, after which the invasive cells on the lower surface of the membrane were stained, dissolved with 10% acetic acid, and transferred to a 96-well plate and optical density (OD) measured at 560 nm.

## Supplementary Information

**Additional file 1: Table S1.** Table of specimens used in study, patient clinical data, and tumor mutational profiles.

**Additional file 2: Table S2.** PCA loadings of AS events described in Fig. 1.

**Additional file 3: Supplementary figures.** Supplementary figures and legends.

**Additional file 4: Table S3.** GBM-specific neojunctions derived from public data analysis.

**Additional file 5: Table S4.** Differential splicing test between all in-house GBM RNA-seq vs. non-malignant brain RNA-seq.

**Additional file 6: Table S5.** Annotated lists of GBM-specific neojunctions derived from in-house data analysis.

**Additional file 7: Table S6.** Putative neojunction-derived neoantigens and HLA serotypes.

**Additional file 8: Table S7.** Differential splicing test between primary and recurrent in-house GBM RNA-seq.

**Additional file 9: Table S8.** Differential expression test between primary and recurrent in-house GBM RNA-seq.

**Additional file 10.** Review history.

## Data Availability

The study data, in the form of raw sequenced reads, are available from the European Genome-phenome Archive repository (https://www.ebi.ac.uk/ega/home), accession EGAS00001004524 [28]. Processed data are also available from the Gene Expression Omnibus repository (https://www.ncbi.nlm.nih.gov/geo/), accession GSE155434 [29]. The third-party data used in this study were paired longitudinal GBM RNA-seq data from TCGA [30] and from INCB [31], as well as GBM mass-spectrometry data obtained from CPTAC [32], and non-malignant brain data obtained from GTEx [33].
