# Supplementary material for: The evolution of alternative splicing in glioblastoma under therapy
Source: Genome Biol. 2021 Jan 26;22:48. doi: 10.1186/s13059-021-02259-5 (PMC7835670; doi:10.1186/s13059-021-02259-5)
Supplement: Supplementary file 2 — Additional file 2: Table S2. PCA loadings of AS events described in Fig. 1. [file 13059_2021_2259_MOESM2_ESM.pdf]

Supplementary Figures:

Fig. S1: Examples of tumor-specific AS events in genes associated with GBM malignant progression.

Fig. S2: A) Putative neojunctions derived from Kahles et al. [2], validated in 10X Genomics scRNA-seq of neoplastic cells. B) The distribution of tumor-specific neonjunctions, derived from analysis of in-house data, compared between primary and recurrent GBM.

Fig. S3: A) Examples of AS events enriched in recurrent-GBM. B) The frequencies of expression of neojunction sequences supporting the inclusion of exon 19 in scRNA-seq of neoplastic cells from human GBMs via the 10X Genomics platform.

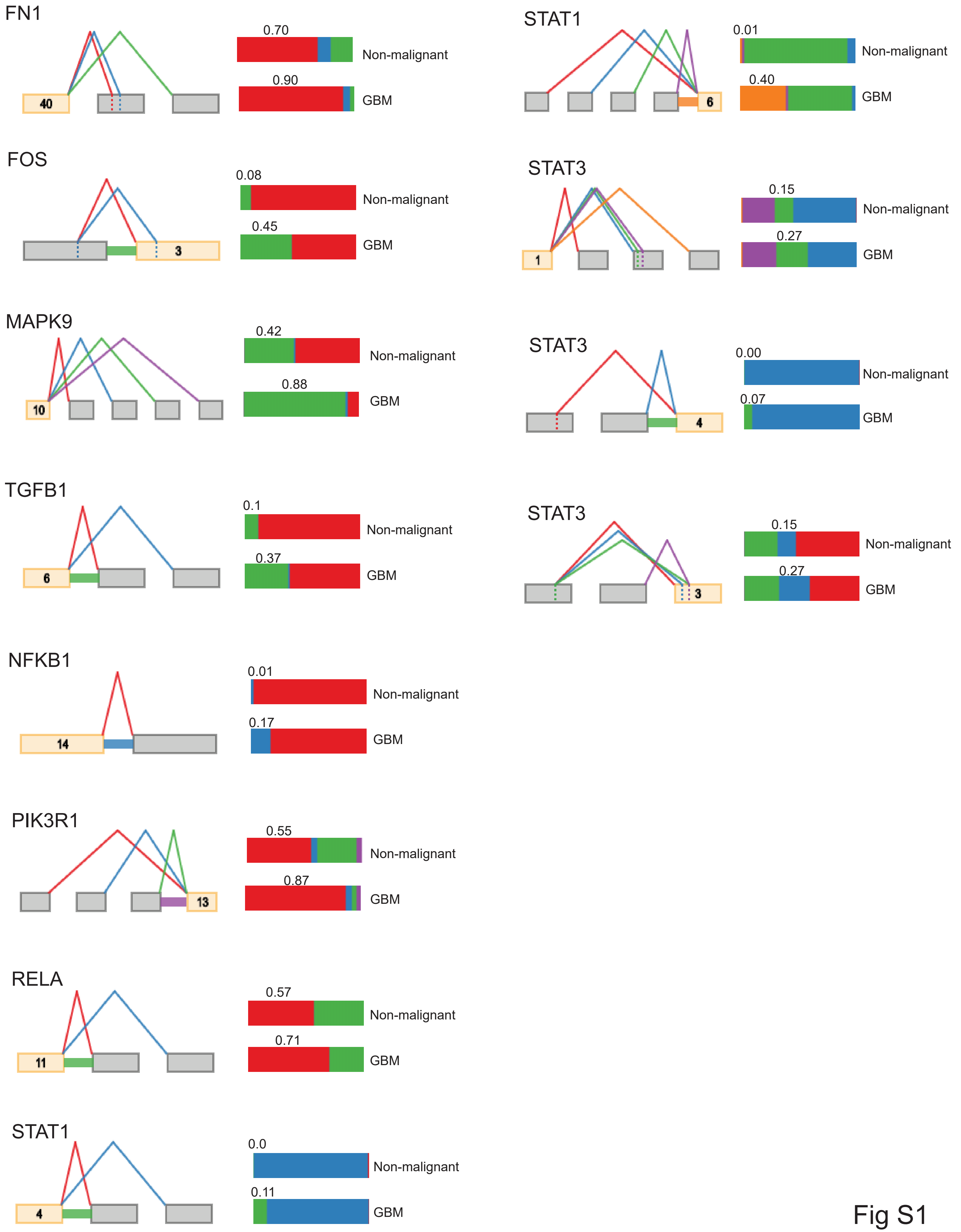
FN1
40
0.70
Non-malignant
0.90
GBM
FOS
3
0.08
Non-malignant
0.45
GBM
MAPK9
10
0.42
Non-malignant
0.88
GBM
TGFB1
6
0.1
Non-malignant
0.37
GBM
NFKB1
14
0.01
Non-malignant
0.17
GBM
PIK3R1
13
0.55
Non-malignant
0.87
GBM
RELA
11
0.57
Non-malignant
0.71
GBM
STAT1
4
0.0
Non-malignant
0.11
GBM
STAT1
6
0.01
Non-malignant
0.40
GBM
STAT3
1
0.15
Non-malignant
0.27
GBM
STAT3
4
0.00
Non-malignant
0.07
GBM
STAT3
3
0.15
Non-malignant
0.27
GBM

Fig S1

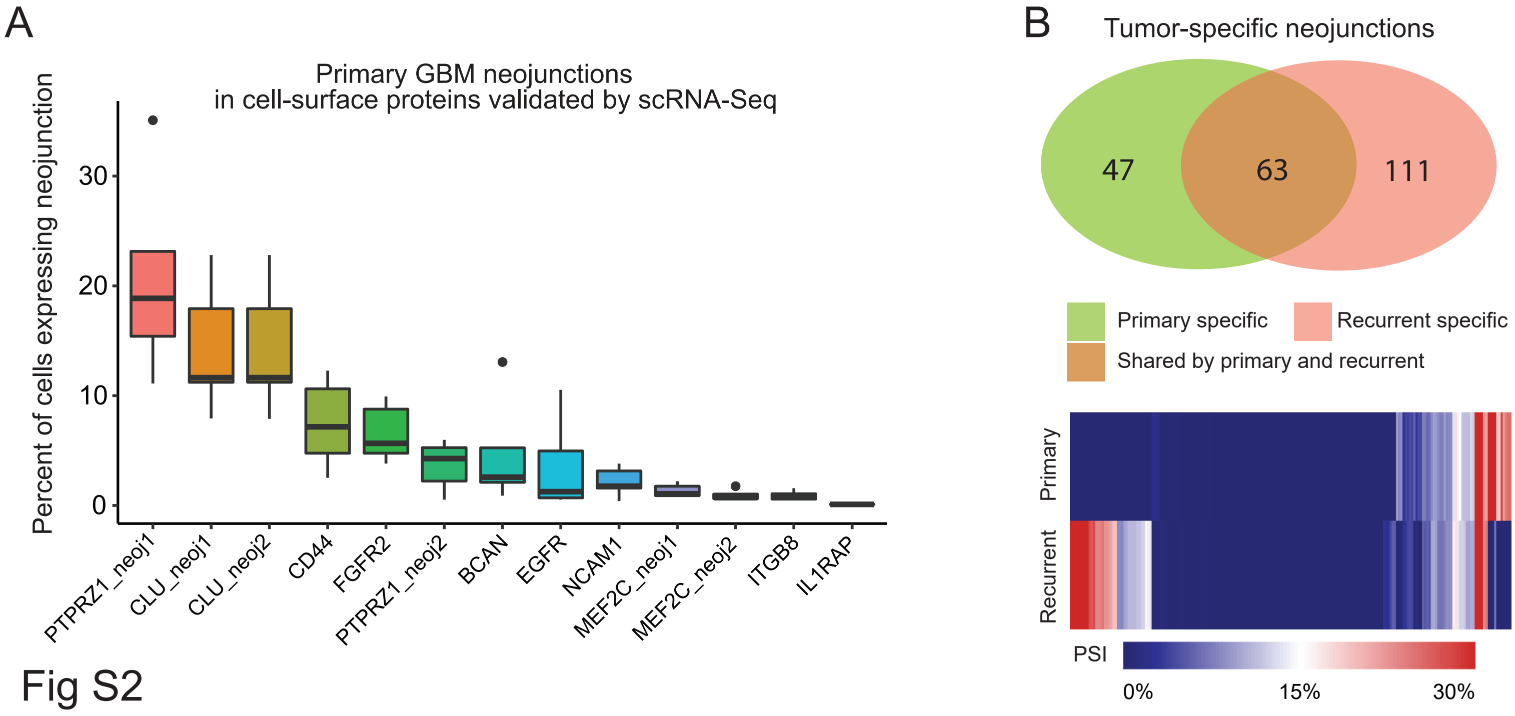
A
Primary GBM neojunctions
in cell-surface proteins validated by scRNA-Seq
Percent of cells expressing neojunction
0
10
20
30
PTPRZ1_neoj1
CLU_neoj1
CLU_neoj2
CD44
FGFR2
PTPRZ1_neoj2
BCAN
EGFR
NCAM1
MEF2C_neoj1
MEF2C_neoj2
ITGB8
IL1RAP
B
Tumor-specific neojunctions
47
63
111
Primary specific
Recurrent specific
Shared by primary and recurrent
Primary
Recurrent
PSI
0%
15%
30%

Fig S2

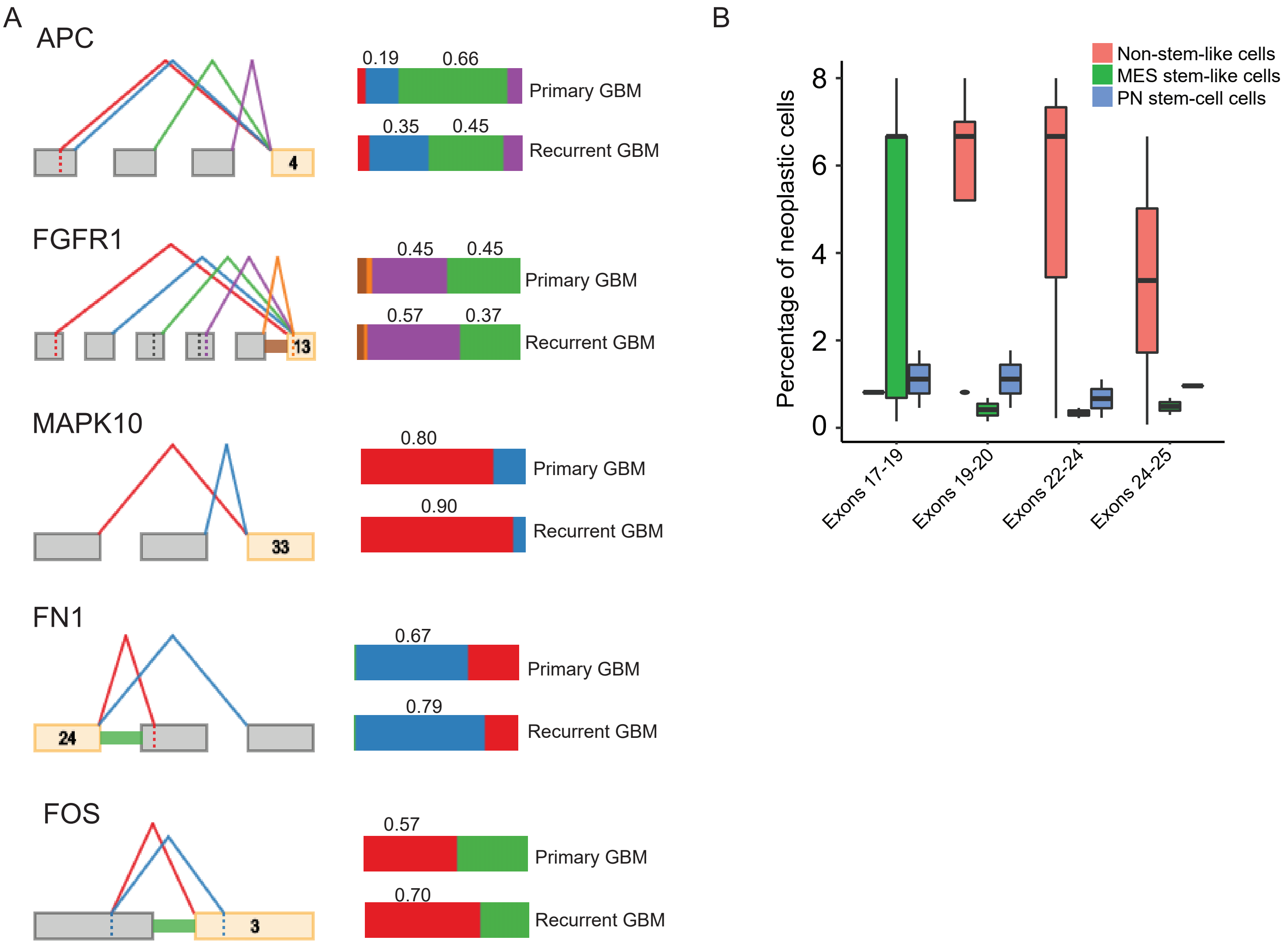
A
APC
0.19
0.66
Primary GBM
0.35
0.45
Recurrent GBM
4
FGFR1
0.45
0.45
Primary GBM
0.57
0.37
Recurrent GBM
13
MAPK10
0.80
Primary GBM
0.90
Recurrent GBM
33
FN1
0.67
Primary GBM
0.79
Recurrent GBM
24
FOS
0.57
Primary GBM
0.70
Recurrent GBM
3
B
Non-stem-like cells
MES stem-like cells
PN stem-cell cells
Percentage of neoplastic cells
8
6
4
2
0
Exons 17-19
Exons 19-20
Exons 22-24
Exons 24-25

Fig S3
