# Supplementary material for: The evolution of alternative splicing in glioblastoma under therapy
Source: Genome Biol. 2021 Jan 26;22:48. doi: 10.1186/s13059-021-02259-5 (PMC7835670; doi:10.1186/s13059-021-02259-5)
Supplement: Supplementary file 10 — Additional file 10. Review history. [file 13059_2021_2259_MOESM10_ESM.docx]

Review History

**First round of review**

**Reviewer 1**

**Are you able to assess all statistics in the manuscript, including the appropriateness of statistical tests used?** Yes, and I have assessed the statistics in my report.

**Comments to author:**

In this manuscript, the authors performed a splicing analysis in primary and recurrent GBMs as well as non-malignant brain tissues through both bulk and single cell RNAseq. The authors identified recurrent-specific MAP4K4 isoforms that are preferentially expressed in mesenchymal stem cells. Moreover, they identified hundreds of neojunction events that is tumor cells specific, providing potential new targets for CART therapy against GBMs. This is a potentially interesting study with potential significance. However, the study is poorly designed with discrete sections. The bioinformatic method is not clearly described. The analyzed results that the authors presented provide minimal valuable information. The current enthusiasm for this manuscript is very low.

1. In Figure 1D, the label of X-axis is missing. The authors should also include information of p-value from enrichment analysis for each pathway.

2. In the second section of Results, the authors identified 172 AS events in 107 genes when comparing primary and recurrent specimens. However, the authors only used the AS in MAP4K4 as an example. More examples should be provided to further support the conclusion. Is there any AS event that has known function in tumor development? Or any AS events in critical tumor-related genes? How was the pathway enrichment analysis of these genes was performed?

3. In Figure 2B, the authors scanned for putative binding sites of RNA-binding proteins across the whole mRNAs of differentially spliced genes, which does not make sense. Instead they should scan for RNA-binding protein motif around the differentially spliced exons.

4. In Figure 2E, the authors should indicate the exon numbers of MAP4K4 that was presented.

5. Figure 2F is inconsistent with the description in the page 4, line 16-18, "over 5-fold increase". Moreover, the label of Y-axis is "Cell percentage". Does it mean the percentage of single cells that show exon 19 included junction reads? The authors should describe their bioinformatic analysis with sufficient details.

6. In the third section of Result, the author should provide sufficient examples of the cancer-specific neojunction AS events. What are their constitutions? What neo-peptide do these neojunction AS events generate? What is the potential of these neo-peptide to be processed as tumor antigen?

7. The section four of Results should be incorporated with section one, since they both analyzed the differentially spliced AS events between malignant and non-malignant samples, as well as primary and recurrent samples.

**Reviewer 2**

**Are you able to assess all statistics in the manuscript, including the appropriateness of statistical tests used?** No, I do not feel adequately qualified to assess the statistics.

**Comments to author:**

In the manuscript entitled "The evolution of alternative splicing in glioblastoma under therapy", Wang and the colleagues characterized primary and recurrent GBM specimens through RNA sequencing to present alternative splicing events in GBM. While the study is of interest, the authors need to demonstrate whether these AS events are highly frequent using larger external validation cohorts. There are numerous studies that have been published that provide such resources that the authors could utilize (PMID: ). Furthermore, there are several overstatements in the manuscript that lack direct supporting evidences such as CAR-T therapy and invasive kinetics. Also there is a lack of methodological details on sample acquisition (which part of the tumor was subjected to RNA sequencing) and analysis. The major comments include:

1. The number of cases that were used in this study is simply too small to draw any concrete conclusions. Furthermore, I believe it is an overstatement to suggest that these AS events are recurrent-tumor specific when there are only limited number of matched primary-recurrent longitudinal pairs. There have been multiple reports that utilize and provide RNA sequencing data from primary-recurrent human GBM specimens. I recommend the authors to use these datasets as external validation to further confirm whether their results are consistent. Furthermore, it would be interesting to see whether which of the AS events are conserved from primary to recurrent and which of them are lost during tumor relapse.

2. There has been a substantial number of studies that employ EGFRvIII as a therapeutic target in terms of CAR-T therapy, owing to its high prevalence in GBM and tumor-specificity. Therefore, I am yet to be convinced whether these other AS events are more ideal targets compared to EGFRvIII.

3. In single cell analysis, what is the total number of cells that were analyzed and among them, how many cells actually had enough coverage to determine MAP4K4 splicing events. Furthermore, how many reads did the authors determine were sufficient to call it as splicing event and their relative ratio to wild-type. These information are not present in the manuscript. Overall, there is a serious lack of methodological section.

4. Distribution of neojunctions in tumor-derived cells in figure 3B between neoplastic cells and glia/immune/endothelial cells does not look significant. Shouldn't "neojuntions in tumor-derived cells" only found in neoplastic cells and not in non-neoplastic cells? Also, typo of "endohelial".

5. The overall theme until figure 3 was focused on identification of new therapeutic approach for recurrent GBMs, as "most clinical trials enroll patients at recurrent" (Abstract). However, this suddenly changes in figure 3 where most of the AS analysis were done using primary GBM specimens when the authors specifically searched for AS events that were highly enriched in recurrent tumors. Then the authors shift back to focusing on recurrent tumor-specific AS events in figure 4. The manuscript needs to show consistency in terms of presenting their argument.

6. The authors need to explain in details what the PSI mean in figure 4C. For those who are not familiar with such concept, it's difficult to follow what the figure represents.

7. Besides, MAP4K4, were there any other AS events that were highly specific to recurrent tumors or primary?

8. The authors mention AS events as potential CAR T-cell targets, but there isn't any supporting evidence, at least in this manuscript, that recognize such claim.

9. The authors propose that they identified recurrent-specific isoforms of mitogen-activated kinase pathways genes that enhance invasiveness, but there are no experimental validations to make such claims.

**Authors Response**

**Point-by-point responses to the reviewers’ comments:**

We thank the reviewers for the constructive critiques. Reviewer comments have helped guide our work. We address reviewer comments with additional invasion assays, increased sample size, bioinformatics analyses and integration of public data. Please find our point-by-point responses below.

There are limited RNA-seq of paired specimens from human glioblastomas available for longitudinal studies. To our knowledge, our novel data represent the largest public cohort of paired-end RNA-seq from longitudinal specimens to date (Table A), representing decades of biobanking at UCSF. Paired-end sequencing is necessary for de novo alternative splicing discovery.

Table A

| ID | Number of samples | Paired-end vs. single-end | Publication |
| --- | --- | --- | --- |
| **This study** | **37** | **29 Paired-end, 8 single-end** | **-** |
| SMC | 42 | *Single-end* | Wang Nat Genet 2016 |
| INCB | 9 | Paired-end | Wang Nat Genet 2016 |
| Nejo | 8 | Paired-end | Nejo CIR 2019 |
| TCGA | 6 | Paired-end | Wang Nat Genet 2016 |

Reviewer 1

- In Figure 1D, the label of X-axis is missing. The authors should also include information of p-value from enrichment analysis for each pathway.

We have corrected this error.

- In the second section of Results, the authors identified 172 AS events in 107 genes when comparing primary and recurrent specimens. However, the authors only used the AS in MAP4K4 as an example. More examples should be provided to further support the conclusion. Is there any AS event that has known function in tumor development? Or any AS events in critical tumor-related genes? How was the pathway enrichment analysis of these genes was performed?

In the revised manuscript, we now highlight additional AS events (Figure 5 and Figure S3). These include AS events in genes critical for malignant progression. This includes , several mitogen-activated protein kinases (*MAP4K4*, *MAPK9*, *MAPK10*), growth-factor receptors (*FGFR1*, *FGFR2*, *EGFR*), and matricellular proteins implicated in invasion (*TNC*, *FN1*). We have now clarified the pathway enrichment approach and parameters in our revised methods section.

- In Figure 2B, the authors scanned for putative binding sites of RNA-binding proteins across the whole mRNAs of differentially spliced genes, which does not make sense. Instead they should scan for RNA-binding protein motif around the differentially spliced exons

Respectfully, we disagree here. Our understanding is that many RNA-binding proteins regulate distal splicing events, through manipulation of RNA secondary/tertiary structure and other means (e.g. Yamazaki RNA 2019, Lovci Nat Struc Mol Biol 2013). We therefore felt that it was important to scan the entire gene and that it was useful to present this additional information.

- In Figure 2E, the authors should indicate the exon numbers of MAP4K4 that was presented.

Fixed.

- Figure 2F is inconsistent with the description in the page 4, line 16-18, "over 5-fold increase". Moreover, the label of Y-axis is "Cell percentage". Does it mean the percentage of single cells that show exon 19 included junction reads? The authors should describe their bioinformatic analysis with sufficient details.

We’ve fixed the typo. We apologize for the confusing axis labels. Here “Cell percentage” refers to a percentage taken out of all neoplastic cells. The x-axis labels refer to junction-spanning reads for the exons indicated. We want to compare the frequency of exon 19 inclusion across neoplastic cell types. These percentages indicate that exon 19 is spliced in for most stem-like cells, which themselves constitute roughly 10% of the neoplastic cells. This further shows that exon 19 is more frequently expressed in stem-like cells of the Verhaak-mesenchymal phenotype, compared to the proneural phenotype. We have revised the methods section considerably to increase the level of detail in description of our bioinformatics approaches.

- In the third section of Result, the author should provide sufficient examples of the cancer-specific neojunction AS events. What are their constitutions? What neo-peptide do these neojunction AS events generate? What is the potential of these neo-peptide to be processed as tumor antigen?

In the revised manuscript we have now provided additional examples of cancer-specific neojunction AS events (Figure 5, Figure S1, and Figure S3). We describe their constitutions in Figure 1E. Our original goal was to elucidate targets for CAR-T therapy. CAR-T cells can be engineered to target any surface antigen and therefore suitable targets are exempt from the criterion which the Reviewer raises, that the antigen be the product of proteolytic processing and HLA-presentation. The Reviewer’s points would be relevant for TCR-transduced T-cell or cancer-vaccine therapies, which were not our original goal.

The Reviewer raises the intriguing possibility of extending our study to identify targets for these other therapies. In the revised manuscript, we have additionally performed HLA serotyping, proteolytic cleavage prediction and class-I HLA binding affinity prediction. We find an overall increase in the number of neojunction-derived antigens with strong HLA affinity and an average increase in the predicted affinities at recurrence (page 4, Figure 4, Table S6).

- The section four of Results should be incorporated with section one, since they both analyzed the differentially spliced AS events between malignant and non-malignant samples, as well as primary and recurrent samples.

To address the Reviewer’s point we moved the sections on autologous T-cell target identification to be subsequent to section one. Hopefully, this will improve the logical progression of the manuscript.

Reviewer 2

- The number of cases that were used in this study is simply too small to draw any concrete conclusions. Furthermore, I believe it is an overstatement to suggest that these AS events are recurrent-tumor specific when there are only limited number of matched primary-recurrent longitudinal pairs. There have been multiple reports that utilize and provide RNA sequencing data from primary-recurrent human GBM specimens. I recommend the authors to use these datasets as external validation to further confirm whether their results are consistent. Furthermore, it would be interesting to see whether which of the AS events are conserved from primary to recurrent and which of them are lost during tumor relapse.

Regarding sample size and impact of the novel data, in the revised manuscript we now include 37 patient-matched longitudinal specimens. There are limited RNA-seq of paired specimens from human GBM (WHO 2016 criterion) available for longitudinal studies. To our knowledge, our novel data represent the largest public cohort of paired-end RNA-seq from longitudinal human GBM specimens to date (Table A), representing decades of biobanking at UCSF for this rare disease. Paired-end sequencing is necessary for de novo alternative splicing discovery. Unfortunately, there are only 23 other publicly available longitudinal specimens with paired-end RNA-seq, as far as we can tell.

To address the Reviewer’s concerns, and validate our findings with available public data (INCB and TCGA datasets) in the revised manuscript, we map public RNA-seq to neojunctions derived from the analysis of our in-house data. These novel validation studies are found in Figure 5B and Figure 6F-G.

We applied for access to the Nejo et al. data, but have not yet received approval for access as of the deadline we were given for revisions. As for SMC data, even though these are only single-end sequencing data and will have low sensitivity for AS analysis, we would have liked to check them as well. However, we have attempted on multiple occasions to contact the data access committee members for the EGA repository for these data and have not received a response as of our six-week deadline for revisions. We acknowledge the limitation in the current sample size (n=52, 37 in house + 15 public) in the discussion section. Despite this limitation we feel the study does contribute novel valuable data and analysis.

- There has been a substantial number of studies that employ EGFRvIII as a therapeutic target in terms of CAR-T therapy, owing to its high prevalence in GBM and tumor-specificity. Therefore, I am yet to be convinced whether these other AS events are more ideal targets compared to EGFRvIII.

Unfortunately, the EGFRvIII variant only occurs in approximately 18% of patients. Moreover, the alteration is often sub-clonal. Thus, there is a critical need to identify additional targets for CAR-T cell therapy.

Co-author Dr. Hideho Okada has been instrumental in the development of EGFRvIII CAR-T therapeutics (e.g. O’Rourke Sci Transl Med 2017, Ohno J Immunother Cancer 2013). Our position is that it is not a matter of identifying a better target than EGFRvIII, but rather complementary targets to provide a more complete treatment for more patients. Our vision is a library of reagents which would enable a precision immunotherapy approach.

- In single cell analysis, what is the total number of cells that were analyzed and among them, how many cells actually had enough coverage to determine MAP4K4 splicing events. Furthermore, how many reads did the authors determine were sufficient to call it as splicing event and their relative ratio to wild-type. These information are not present in the manuscript. Overall, there is a serious lack of methodological section.

We apologize for any omissions in the methodology section in the original manuscript. We have significantly revised this section. As for quantifying MAP4K4 splicing events in single-cell data, we did not call splicing events in single cells. We only used deeply sequenced bulk-RNA sequencing data for de novo AS discovery. Rather we quantify expression of neojunction sequences in single cells by mapping reads from single-cell RNA-seq data to a reference comprised of neojunctions that were derived from bulk-RNA sequencing analysis. The number of cells which express a given neojunction sequence are then reported. We interrogated 3873 neoplastic cells from 9 patients for this analysis.

- Distribution of neojunctions in tumor-derived cells in figure 3B between neoplastic cells and glia/immune/endothelial cells does not look significant. Shouldn't "neojuntions in tumor-derived cells" only found in neoplastic cells and not in non-neoplastic cells? Also, typo of "endohelial".

Apologies for the confusion, we have changed the panel’s title to “… putative neojunctions in GBM scRNA-seq”. Figure 3B (now Figure 2B) does not describe the overlap or difference in expression between neoplastic/non-neoplastic cells. This panel shows the percentages of putative neojunctions (estimated from bulk RNA-seq tumor/normal comparisons) that were positively validated in neoplastic scRNA-seq, or negatively filtered because they were found in scRNA-seq of non-neoplastic cells. Subsequent panels Figure 3C-D (now Figure 2C-D) and Table S3 contain only putative neojunctions that are validated via scRNA-seq as expressed in neoplastic cells and not expressed in non-neoplastic cells.

We have fixed the typo.

- The overall theme until figure 3 was focused on identification of new therapeutic approach for recurrent GBMs, as "most clinical trials enroll patients at recurrent" (Abstract). However, this suddenly changes in figure 3 where most of the AS analysis were done using primary GBM specimens when the authors specifically searched for AS events that were highly enriched in recurrent tumors. Then the authors shift back to focusing on recurrent tumor-specific AS events in figure 4. The manuscript needs to show consistency in terms of presenting their argument.

We appreciate this comment and have moved the analysis of MAP4K4 and other AS events enriched at recurrence to the end of the manuscript. Hopefully, this will provide a more logical flow for the manuscript, along the lines the Reviewer recommends.

- The authors need to explain in details what the PSI mean in figure 4C. For those who are not familiar with such concept, it's difficult to follow what the figure represents.

To address this we have added an explanation of the meaning of PSI to the main text (page 3).

- Besides, MAP4K4, were there any other AS events that were highly specific to recurrent tumors or primary?

Yes, we found multiple recurrent specific AS events in genes associated with malignant progression. We have detailed some of these examples in Figure 5A and Figure S3.

- The authors mention AS events as potential CAR T-cell targets, but there isn't any supporting evidence, at least in this manuscript, that recognize such claim.

We have identified splicing alterations in cell-surface genes that occur in extracellular domains, and that are specific to neoplastic cells compared to non-malignant glia. These are excellent preliminary candidates as far as RNA-level information can provide. Subsequent efforts will be required to validate these candidates for use in CAR-T. Firstly, we must identify antibodies that are specific to the cancer-enriched isoforms. We have previously used phage-library screens for this purpose, which is a lengthy process for each candidate. Once an antibody is identified there are then many steps to transduce CAR-T cells to express this antibody and to validate that the resulting CAR-T cells recognize the target antigen and engage downstream cytotoxic effector function. We feel that this work is beyond the scope of the current manuscript. Moreover, additional assays would have been challenging given the six-week deadline provided to us for revisions. We have included in the discussion the need for additional validation studies as a limitation of the current study, and we have attempted to soften our claims throughout along the lines suggested by the Reviewer’s point.

- The authors propose that they identified recurrent-specific isoforms of mitogen-activated kinase pathways genes that enhance invasiveness, but there are no experimental validations to make such claims.

In the revised manuscript, we address this with additional invasion assays. We transfected U87 glioblastoma cells with plasmids expressing *SRSF5* or an empty-vector control. Following selection, we performed extra-cellular matrix invasion assays, with and without temozolomide treatment. We found that *SRSF5* over-expression enhanced invasion, and this effect was exacerbated by temozolomide (GBM standard-of-care; Figure 6I).

Our efforts were limited by the six weeks given to us for revisions. Many important assays were left undone, e.g. validation of differential SRSF5 binding, SRSF5 Western blots, differential MAP4K4 exon-19 inclusion, elucidation of downstream effector genes and validation in vivo. Nonetheless, this finding supports to the hypothesis that dysregulated splicing contributes to glioma malignancy and response to therapy.

**Second round of review**

**Reviewer 1**

The authors addressed most of my critiques. However, it is imperative that the authors address the following two major concerns with supporting results:

1. The authors provided more examples of recurrent-specific AS events in Fig.5 and Fig. S3. However, the authors should validate their bioinformatic analysis by experimental data. They should design primers and perform RT-PCR assay to detect the splicing pattern of at least 5 candidates in clinical specimens of primary/recurrent GBM and normal brain tissues.

2. The evidence for the identified cancer-specific neojunction AS events as potential CART cell target is still insufficient. The authors could analyze publically available proteomic data of GBM, such as CTPAC database, https://proteomics.cancer.gov/programs/cptac, to confirm the existence of neo-peptides generated from their identified neojunction AS events and determine their frequency across all GBM samples.

**Reviewer 2**

In their revised version, the authors have addressed my comments to the best ability within the limits of the data. The revised version represents a balanced presentation of the data and I believe will be of interest to the field.

**Authors Response**

**Point-by-point responses to the reviewers’ comments:**

Thank you for your continued interest in our manuscript. We were gratified to read that Reviewer #2 is satisfied with our additional assays and believes the manuscript is of interest to the field. We were also glad to hear Reviewer #1 say: “The authors addressed most of my critiques.”. In our opinion, this study is a significant advance in several regards:

1.	There are limited RNA-seq data from longitudinal human GBM specimens. To our knowledge, our novel data represent the largest cohort of paired-end RNA-seq from longitudinal GBM specimens to date, requiring decades of biobanking at UCSF.

2.	We report cell type-specific splicing in GBM at recurrence, for the first time.

3.	We identify recurrent-specific isoforms of mitogen-activated kinase pathway genes that enhance invasiveness and are preferentially expressed by glioma stem-like cells. We determine the upstream regulator to be aberrant expression of splicing factor SRSF5 and that SRSF5 overexpression enhances the invasion of extracellular matrix by glioma cells.

4.	We identify novel splicing events in cell-surface proteins that are suitable targets for development as engineered T-cell therapies. This is an exciting emerging therapeutic modality and a lack of specific, putative targets for CAR T-cell therapy development is a bottleneck and represents a critical need.

Reviewer #1 makes novel critiques which were not raised in the initial review. In our revised manuscript, we have now addressed Reviewer #1’s request that we compare our findings to the CPTAC database (summarized below). However, regarding the reviewer’s second request that we perform an RT-PCR assay to validate alternative-splicing events observed in our RNA-seq, we must respectfully decline. Our reasoning is the following:

1.	It is not clear that this validation is essential. For example, Kahles Cancer Cell 2018 performs similar splicing analyses as ours (they don’t have longitudinal specimens) and they don’t do any PCR validation.

2.	It is unclear what type of RT-PCR the reviewer is referring to, semi-quantitative or quantitative. If the reviewer is referring to quantitative RT-PCR, then primer design and optimization could potentially take months and cost many thousands of dollars, as we must optimize primers to anneal the splice junctions for each AS event. This assay will require a minimum of five custom-order primers per gene, and potentially many more custom primers will need to be generated during the optimization process. On the other hand, if the reviewer is referring to semi-quantitative RT-PCR then there is not a need for such precision in primer design. However, for semi-quantitative RT-PCR the readout is an immunoblot which has a very low throughput. So, in that case we would only be able to go through a handful of clinical specimens, which would not be much of a validation. Moreover, optimizing the immunoblots would be very time consuming since the molecular weights of the variant isoforms will be similar.

3.	It is unclear how many clinical specimens the reviewer expects us to perform validations on. This is related to the previous point, since in order to validate a cohort of any significant size we would need to do quantitative RT-PCR.

4.	Lastly, the reviewer requests that we do RT-PCR from non-malignant brain tissue. However, ethically-sourced non-malignant tissue from adult human brain is exceedingly rare as it is only resected during the course of specific epilepsy surgeries. Fetal brain tissue (which is also very limited) would not be a suitable alternative control. We are unable to obtain such rare specimens in the case numbers needed for a control within a reasonable time frame.

For these reasons, we will not be able to perform these studies within the time frame allocated for revisions. We do not wish to delay publication of these novel data and results.

We have chosen to focus on the Reviewer’s first request, that we compare our findings to the CPTAC proteomics database. We followed the approach of Kales Cancer Cell 2018 and used OpenMS to identify polypeptides derived from our neojunctions. We found that over 75% of our patient samples express at least one CPTAC-validated putative CAR-T target, with three-to-four neojunctions confirmed per sample on average (Figure 3E-F; Table S5).

We consider this to be a conservative underestimate since alternative splicing-derived junction-spanning polypeptides are poorly represented in mass-spectrometry data such as CPTAC due to the cleavage properties of trypsin, see Wang et al. Detection of proteome diversity resulted from alternative splicing is limited by Trypsin cleavage specificity. Mol Cell Proteomics. 2018. Note that the CPTAC database cannot be used to assess a neoantigen’s population frequency as the methodological approach to generating the CPTAC glioblastoma data involved pooling glioblastoma specimens. In particular, 110 glioblastoma specimens were pooled in cohorts of 10, to yield 11 analytical samples.
